# How to detect myopia in the eye clinic

**Published:** 2019-05-13

**Authors:** Michelle L Hennelly

**Affiliations:** 1 MSc Programme Director in Clinical Optometry: Division of Optometry and Visual Science, City, University of London, UK

**Ophthalmic nurses and other allied health personnel can detect myopia using a Snellen chart and a pinhole occluder.**

**Figure:**
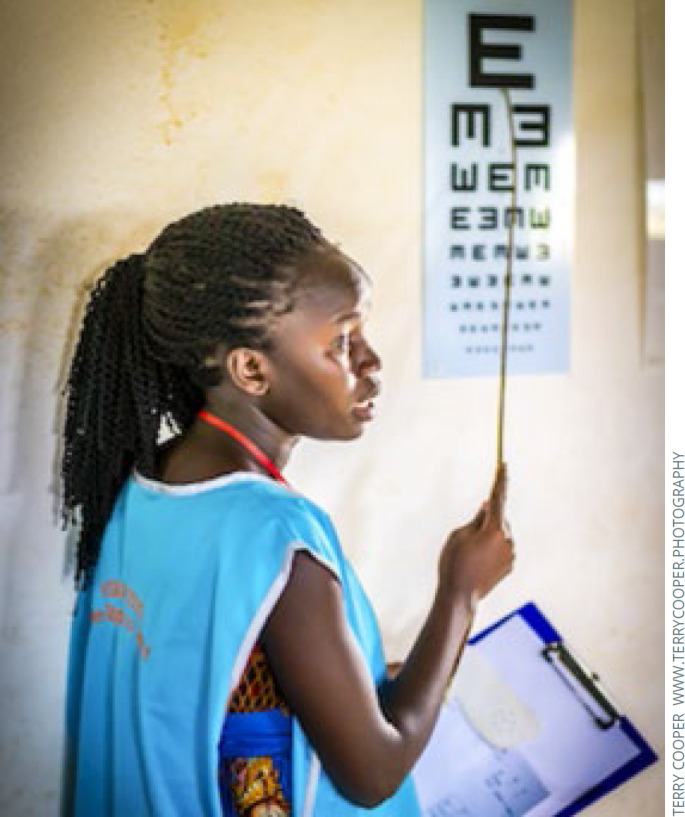
Testing visual acuity using a tumbling E chart. UGANDA

Myopia is a refractive error that occurs when the eye is longer than normal or has a cornea which is too steep (p. 4). People with myopia, also known as short- or near-sightedness, can see near objects clearly, but objects further away appear blurred and out of focus. Normal vision can be restored by prescribing the correct spectacles or contact lenses.

Ideally, refractive errors in children (myopia, hypermetropia and/or astigmatism) should be detected in eye screening programmes in schools or in the community. In the absence of such programmes, adults and children will present at the clinic with a range of conditions.

It is therefore important to be aware of myopia and to look out for it when performing an eye examination.

## About myopia

Myopia can be inherited, and there is also evidence that it is more likely to occur in communities where children spend less time outdoors and more time doing near work. It can be corrected using spectacles or contact lenses of the correct power, expressed in dioptres (D). People who require ≤ −0.50 D of optical correction are considered as having myopia.

**“There is evidence that myopia is more likely to occur in communities where children spend less time outdoors and more time doing near work.”**

High myopia affects around 10% of people with myopia, and is defined by the World Health Organization as requiring ≤ −5 D of correction. People with high myopia are at greater risk of macular degeneration, retinal detachment, glaucoma and cataract. Of these, cataract is the only reversible condition: sight can be restored in a single, quick surgical procedure. Retinal detachment can cause sudden visual loss and requires urgent surgical treatment to re-attach the retina; failure to do so can result in a complete loss of vision in that eye. Macular degeneration and glaucoma cause progressive visual loss that cannot be reversed, so early detection is essential as medication or surgery can, at best, slow down or halt their progression.

This article describes how to detect myopia and when to refer someone to the appropriate health professional for a comprehensive eye examination, refraction and spectacle prescription.

## 1 History, symptoms and signs

Indicators of myopia in children include:

- Poor distance vision
- Viewing objects from an unusually short distance
- Poor concentration in school
- Squinting or peering though narrowed eyes.

People with myopia may complain of:

- Blurred distance vision
- Frontal headaches.

Ask about the person's previous eye history, e.g. spectacles or lazy eye, and about family history, including myopia (due to the familial link), glaucoma, diabetes mellitus and hypertension. Floaters and flashes of light are associated with retinal detachment and requires urgent referral to an ophthalmologist. Double vision is associated with a range of conditions, including strabismus (lazy eye) and neurological disorders, and requires referral to a medical professional or ophthalmologist.

Ask about general health, medications, whether the person drives, and their hobbies.

End with the open–ended question: “Is there anything else that you feel I should know about your eyes or your health?” If you have been thorough in your questioning, the answer should be no, which can be recorded in the notes as NOC (no other comments).

## 2 Measure visual acuity

Visual acuity testing must form part of every eye examination. ‘No refractive error’ at this distance is deemed to be 6/6 or 20/20, even though there may be even smaller lines on the chart, such as 6/5 or 6/4.

### Steps

- Use a Snellen chart, placed 6 metres (20 feet) away from the person.
- For younger children or those who cannot read, use a tumbling E or a tumbling C chart and ask them to point in the direction of the opening in each letter.
- Ensure there is good natural light or illumination on the chart, as Snellen charts are designed to test central vision at high contrast.^1^
- Explain the procedure to the person.
- Position the person, sitting or standing, at a distance of 6 metres (20 feet) from the chart.
- Clean and dry the occluder. If no plain occluder is available, use clean card or a tissue. Ask the patient to cover one eye but not to press on it.
- Test one eye at a time. Starting from the top of the chart, ask the person to read the letters (Snellen chart) or point in the direction of the open end of the letter (tumbling E or C chart). Position the chart at 3 metres (10 feet) if the person's vision is less than 6/60 and record as 3 metres instead of 6 (e.g. 3/60).
- Record the visual acuity (written as a fraction next to the smallest line the person can read). For example, if the person cannot read the bottom row (visual acuity of 6/6) but can read the next row of letters (6/9) then their visual acuity is 6/9.
- If the patient cannot see the letters on the 6/6 line, they may have a refractive error, such as myopia.

## 3 Perform a pinhole test

Pinhole testing is mainly used for adults and older children. Children under 7–8 years old would struggle to see with a single pinhole. Occluders with multiple pinholes may work better, but if these are unavailable, refer all children with VA of < 6/6 for refraction.

A pinhole occluder (an opaque disc with one or more small holes) is used to determine whether reduced vision is caused by refractive error. If this is the case, the pinhole will cause an improvement in visual acuity.

If the pinhole worsens vision, this can indicate macular disease, central lens opacities or other causes of reduced vision. If there is no change in visual acuity, this might be caused by amblyopia. Children and adults suspected of having these conditions must be referred.

**Figure:**
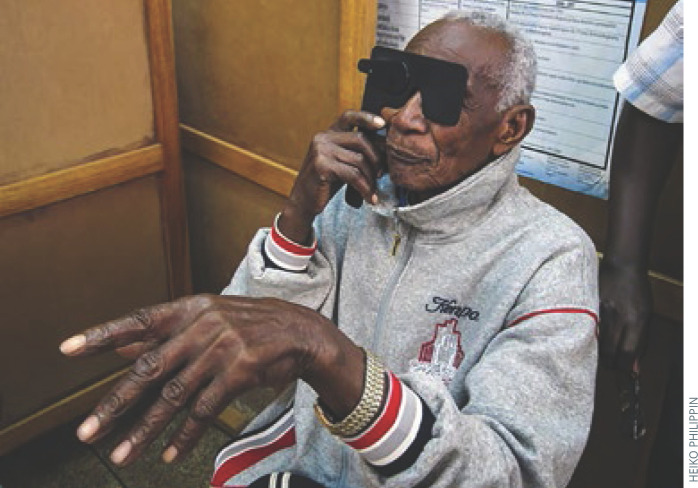
A pinhole occluder is an opaque disc with one or more small holes. In people with myopia, visual acuity increases with the use of a pinhole. TANZANIA

### Steps

- Clean and dry the pinhole occluder.
- Ask the patient to cover one eye with the occluder and position the pinhole so they can see through it.
- Test one eye at a time by following the same procedure used to test visual acuity.

## 4 Should I refer?

If the person can read more letters with the pinhole than without, they are likely to have a refractive error, such as myopia. All patients (adults and children) whose acuity improves with a pinhole, and/or who present with symptoms consistent with a refractive error, should be referred for a full refraction and an eye health examination. Refer patients with signs or symptoms of eye disease for a comprehensive eye examination (including a slit lamp examination, if possible) if you are unable to carry one out yourself.

## References

[1] Marsden J Stevens S Ebri A. How to measure distance visual acuity. Comm Eye Health J 2014;27(85):16. PMC4069781 24966459

